# Supplementary material for: The Role of Technology in Online Health Communities: A Study of Information-Seeking Behavior
Source: Healthcare (Basel). 2024 Jan 29;12(3):336. doi: 10.3390/healthcare12030336 (PMC10855704; doi:10.3390/healthcare12030336)
Supplement: Supplementary file 1 [file healthcare-12-00336-s001.zip › healthcare-2813213-supplementary.pdf]

Supplementary Materials

1. What is your age?
    a. Under 30
    b. 31 - 54
    c. 55 - 64
    d. 65 - 74
    e. Over 75
2. What is your gender?
    a. Male
    b. Female
    c. Other
3. What is your marital status?
    a. Married
    b. Single
    c. Widowed
    d. Divorced

4. What is your annual income?
    a. Less than $49,000
    b. $50,000 - $99,000
    c. More than $100,000
5. What country are you living at this time?
6. What is your cultural background and how does it affect your healthcare decision making?

For each of the questions below, circle the response that best characterizes how you feel about the statement, 1 = Strongly Disagree, 2 = Disagree, 3 = Neutral, 4 = Agree, and 5 = Strongly Agree.

	Strongly disagree	Somewhat disagree	Neither agree nor disagree	Somewhat agree	Strongly agree
1. The thought of COPD scares me.	1	2	3	4	5
2. COPD is a hopeless disease.	1	2	3	4	5
3. I worry about my physical health deteriorating as my COPD progresses.	1	2	3	4	5
4. I have a lot to gain by participating in this forum.	1	2	3	4	5

5. Participating in this forum helps me in controlling my COPD.	1	2	3	4	5
6. I am less anxious about my disease by participating in this forum.	1	2	3	4	5
7. It is convenient for me to participate in this forum.	1	2	3	4	5
8. It is easy for me to share knowledge in this forum.	1	2	3	4	5
9. Participating in this forum does not interfere with my daily routines.	1	2	3	4	5
10. By sharing knowledge in this forum, I pour out my troubles and feel relaxed.	1	2	3	4	5
11. By sharing knowledge in this forum, I get some understanding, help, or support from other participants in the community.	1	2	3	4	5
12. By sharing knowledge in this forum, I get comfort and care from other participants in the community.	1	2	3	4	5
13. Using this forum is useful in managing my COPD.	1	2	3	4	5
14. Using this forum for health information is advantageous in better managing my health.	1	2	3	4	5
15. Using this forum for health information about my COPD is beneficial to me.	1	2	3	4	5

	Strongly disagree	Somewhat disagree	Neither agree nor disagree	Somewhat agree	Strongly agree
16. My interaction in this forum for health information is understandable.	1	2	3	4	5

17. It is easy to navigate to find health information in this forum.	1	2	3	4	5
18. It is easy for me to become skillful at using this forum for health information.	1	2	3	4	5
19. Sharing knowledge enhances my reputation in this forum.	1	2	3	4	5
20. I get praises from others by sharing knowledge in this forum.	1	2	3	4	5
21. Sharing knowledge improves my status in this forum.	1	2	3	4	5
22. Sharing knowledge will help other participants in this forum solve problems.	1	2	3	4	5
23. It is important that members in this forum think positively about me.	1	2	3	4	5
24. Sharing knowledge brings a positive influence on other members in this forum.	1	2	3	4	5
25. I obtain information that is credible in this forum.	1	2	3	4	5
26. I obtain information that is accurate in this forum.	1	2	3	4	5
27. I obtain information that is relevant in this forum.	1	2	3	4	5
28. I obtain information that is reliable in this forum.	1	2	3	4	5
29. I obtain information in a timely manner in this forum.	1	2	3	4	5

30. What about this forum encourages you to join and continue to participate?

31. What is the most valuable asset this forum offers you?

32. How do you evaluate the medical accuracy of the information you are receiving in this forum when you plan to make health decisions for yourself or someone else you care about?
